# In Vitro Accuracy Analysis of Intraoral Scanning Strategies: A Comparison of Two Contemporary IOS Systems

**DOI:** 10.3390/dj14010052

**Published:** 2026-01-13

**Authors:** Sabina-Ana Răuță, Vlad Gabriel Vasilescu, Lucian Toma Ciocan, Alexandra Popa, Ana-Maria Cristina Țâncu, Florin Octavian Froimovici, Bogdan Dimitriu, Silviu-Mirel Pițuru, Marina Imre

**Affiliations:** 1 Discipline of Dental Prosthesis Technology, Faculty of Dentistry, “Carol Davila” University of Medicine and Pharmacy, Dionisie Lupu Street, No. 37, District 2, 020021 Bucharest, Romania; sabina-ana.rauta@drd.umfcd.ro (S.-A.R.); vlad.vasilescu@umfcd.ro (V.G.V.);; 2 Department of Biochemistry, Faculty of Dental Medicine, “Carol Davila” University of Medicine and Pharmacy, 8 Eroilor Sanitari Blvd, 050474 Bucharest, Romania; 3 Discipline of Prosthodontics, Faculty of Dentistry, “Carol Davila” University of Medicine and Pharmacy, 37 Dionisie Lupu Street, District 2, 020021 Bucharest, Romania; anamaria.tancu@umfcd.ro (A.-M.C.Ț.); marina.imre@umfcd.ro (M.I.); 4 Department of Endodontics, Faculty of Dental Medicine, “Carol Davila” University of Medicine and Pharmacy, 8 Eroilor Sanitari Blvd, 050474 Bucharest, Romania; bogdan.dimitriu@umfcd.ro; 5 Department of Organization, Professional Legislation and Management of the Dental Office, Faculty of Dental Medicine, “Carol Davila” University of Medicine and Pharmacy, 37 Dionisie Lupu Street, District 2, 020021 Bucharest, Romania; silviu.pituru@umfcd.ro

**Keywords:** intraoral scanners, digital dentistry, dental impression technique, scanning strategy

## Abstract

**Background**: Digital intraoral scanning has become an essential component of modern restorative dentistry, offering enhanced accuracy, workflow efficiency, and patient comfort compared to conventional impression techniques. Despite these advantages, the accuracy of intraoral scanners (IOS) can be affected by multiple parameters, among which scanning strategy and device design are particularly influential. **Purpose**: This study aimed to investigate the effect of different scanning strategies on scan accuracy and precision, focusing on two widely used intraoral scanners (Medit i700 and Trios 5) in a controlled in vitro environment. **Materials and Methods**: A standardized digital test object was created according to ISO 20896-1 specifications to ensure uniformity and comparability. The object was printed using a high-precision 3D printer and scanned multiple times with both IOS systems, employing distinct scanning strategies under identical environmental conditions. Data analysis was performed using descriptive and comparative statistics, including Mean, Median, Mean Absolute Deviation (MAD), Root Mean Square Error (RMSE), Standard Deviation (SD), and Variance, to evaluate trueness and precision. **Results**: The Medit i700 consistently exhibited lower deviation values and greater precision compared with the Trios 5, reflecting higher trueness and precision. Scanning strategy influenced scan outcomes; structured, systematic scanning paths produced more stable and accurate datasets. The Trios 5 demonstrated higher variability, suggesting increased sensitivity to operator motion and scanning trajectory. **Conclusions**: Both the scanner type and scanning strategy substantially affect intraoral scan accuracy. The superior performance of the Medit i700 indicates greater robustness and operator-independent stability. Clinically, these results underscore the importance of standardized scanning protocols, as operator consistency may be a key determinant of digital impression accuracy and, consequently, of clinical outcomes.

## 1. Introduction

Intraoral scanners (IOS) have been employed in dentistry since the late 20th century [1], emerging as a response to the limitations of traditional impression techniques and conventional materials, which frequently involved dimensional distortions, polymerization shrinkage and discomfort for the patient. The technological evolution of these devices accelerated with the introduction of CAD/CAM (Computer-Aided Design/Computer-Aided Manufacturing) systems, marking the transition toward a fully digital dentistry era [2]. This technological revolution has enabled precise three-dimensional reconstruction of the prosthetic field, facilitating effective communication between clinicians and dental technicians while reducing workflow duration and complexity.

The use of IOS in daily practice offers multiple advantages, including predictable treatment planning, rapid digital communication with the dental laboratory, elimination of the need for physical storage of models, reduction in clinical time, and last but not least, increased comfort for the patient [3,4]. However, to fully harness these benefits, it is essential to understand the internal mechanisms governing IOS operation, as well as the factors that may affect the accuracy of the obtained results. Such factors include scanning strategy, the characteristics of the dental surface, lighting conditions, humidity, and operator movement, each potentially introducing three-dimensional reconstruction errors [5,6,7].

At present, numerous studies have shown that, in the context of fully dentate cases, digital impression techniques can achieve an accuracy comparable to, or even exceeding that of conventional ones [8]. However, the performance of a scanning system is closely dependent on the scanning strategy used, which directly influences parameters such as trueness, accuracy and precision [9]. Although manufacturers provide specific recommendations regarding the ideal scanning path, differences in image acquisition algorithms, sensor resolution, and internal alignment systems result in notable variations between models and even between scanning sessions performed by the same operator.

In this context, the first purpose of this article is to determine the scanning strategy that provides the highest accuracy under controlled conditions (in vitro), using a standardized protocol. The second objective is to evaluate the influence of the scanning system on the performance of each strategy, by comparing results obtained from two widely used scanners in contemporary clinical practice: the Medit i700 and the Trios 5. These two systems were selected as representative of clinically established generations of IOS at the time of study design and data acquisition, and due to their differences in the optical architecture, reconstruction algorithms and automatic calibration strategies.

By correlating these factors, the present research aims to establish the optimal combination of the scanning strategy and the IOS system, ensuring an accurate and predictable geometric reproduction of an ideal digital model–test object. In addition, the comparative analysis of the scanning times and the stability of the values obtained, can provide clinically relevant insights, contributing to the optimization of digital workflows and increasing the efficiency of the operator.

Although most manufacturers recommend their own sets of “tips and tricks” for the optimal use of each scanner [10,11], no clear consensus exists yet, regarding the actual efficiency of various scanning strategies depending on how the scanner works. For example, both manufacturers of intraoral scanners recommend performing the intraoral scan after checking the tip mirror and after calibration, in an extremely dry environment and without additional light from the dental unit. In addition, 3Shape recommends an ideal scanning strategy, which will be described later in this study as the mixed strategy, with linear movements on the posterior teeth and an S-shaped movement on the anterior teeth. The present study provides an essential contribution by directly comparing scanning strategies using the same test object and identical experimental conditions. Consequently, the results obtained may serve as a foundation for clinically relevant recommendations aimed at maximizing accuracy and minimizing operator-induced errors in the digital impression process.

The first null hypothesis of this study posits that the accuracy of intraoral digital impressions is independent of the scanning strategy applied. This hypothesis aims to determine whether different scanning strategies influence the trueness and precision of the final digital impression or whether modern intraoral scanners can compensate for procedural variability through their built-in algorithms. Given that intraoral scanners capture data through successive image stitching and software alignment, variations in scanning path, angulation and sequence may affect the accuracy of the reconstructed master model.

The second null hypothesis asserts that there is no difference in the accuracy performance between the two tested intraoral scanners (Medit i700 and Trios 5) under identical experimental conditions. This assumption investigates whether recent hardware and software developments, in these systems result in measurable improvements in precision or whether both scanners deliver comparable outcomes in controlled in vitro settings. Since both devices employ advanced optical capture and data-processing technologies, evaluating their relative performance is essential to understanding the current state of digital impression accuracy.

By testing these null hypotheses, the study aims to deepen the understanding of how scanner design and scanning strategy interact in determining the fidelity of digital impressions. The outcomes are expected to contribute to the ongoing discussion on optimizing scanning protocols, improving precision, and guiding clinicians in selecting appropriate systems and workflows for achieving reliable intraoral digital impressions.

## 2. Materials and Methods

### 2.1. Reference Master Model

The study used Blender software (Blender v.3.3 LTS, Blender Foundation, Amsterdam, The Netherlands), a versatile and widely used digital tool for 3D modeling [11,12], in order to accurately build a digital master model, hereafter referred to as the master model or the test object. The digital master model has been designed to faithfully reproduce the geometry and specifications defined by the ISO 20896-1 standard, entitled “Dentistry—Digital impression devices—Part 1: Methods for assessing accuracy” [13,14]. The aim of this approach was to ensure compliance with international standards for assessing the accuracy of digital dental impression devices. The ISO standard sets the requirements for digital dental models, with the objective of promoting geometric accuracy and dimensional stability.

In accordance with ISO specifications, the digital test object included four calibration balls (“gauge ball”) with a diameter of 6 mm. Strict adherence to these specifications was essential to maintain consistency and comparability with established test protocols [13,14]. The use of Blender software allowed precise control over the design and dimensions of the digital master model, making it easy to create a faithful replica of the standardized object.

**Figure 1:**
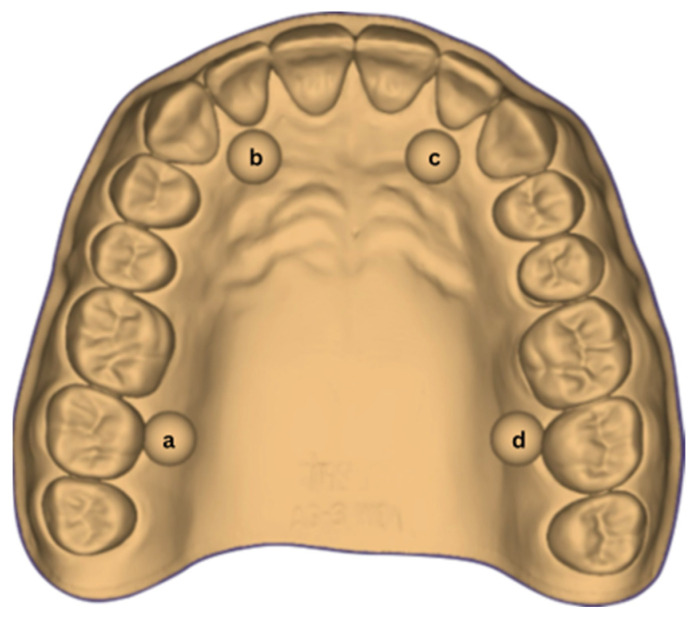
Digital representation of the test object constructed in accordance with ISO standards (a, b, c, d—calibration balls).

Figure 1 illustrates the digital representation of the test object, highlighting its geometry and dimensions. Through rigorous attention to detail and compliance with ISO guidelines, the aim was to achieve a standardized test environment that would allow accurate and reproducible evaluation of the performance of the intraoral scanners analyzed.

The detailed documentation of these aspects ensures transparency and the possibility of replication of the experimental methodology.

The realization of the digital test object according to the ISO standard demonstrates rigor and precision in experimental design (Table 1). This standardized approach forms the basis for objective and meaningful comparisons on the accuracy of the intraoral scanners evaluated, contributing to the advancement of knowledge in the field of digital dentistry.

The dimensions analyzed for the complete dental arch included the six distances (D) defined between the centers of the calibration balls, measured between points ‘a–b’, ‘b–c’, ‘c–d’, ‘d–a’, as well as the diagonals ‘d–b’ and ‘a–c’. This measurement method allows a detailed analysis of both sagittal and transverse dimensions.

The six distances (D), denoted D a–b, D b–c, D c–d, D a–d, D a–c and D b–d, were measured and recorded as reference values (“true values”).

**Figure 2:**
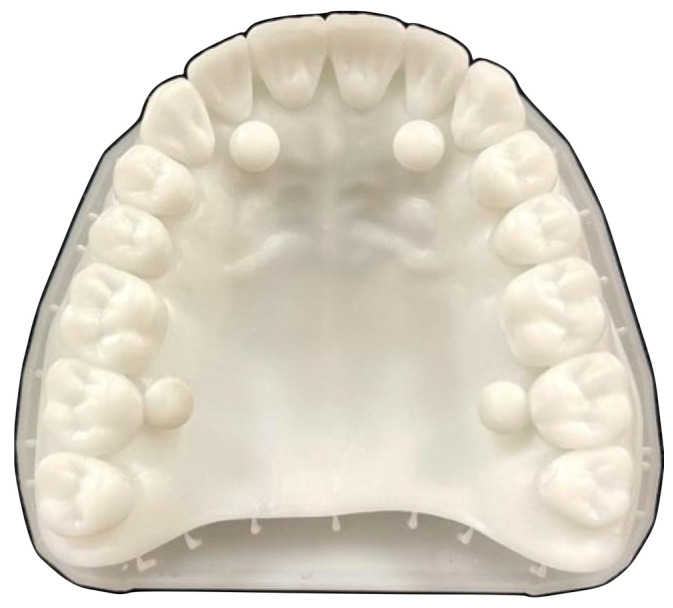
Master model fabricated using the Asiga 3D printer, illustrating the physical replica of the digital test object.

To materialize the test object (Figure 2), the Asiga 3D printer (Asiga model, Sydney, Australia) was used, which allowed the digital master model to be transposed into physical form [14]. This printer, recognized for its accuracy, resolution and operational efficiency, played a key role in the manufacturing process, ensuring a faithful reproduction of the digital master model [15].

By using this technology, standardized replicas of the test object have been obtained, conforming to the specifications of ISO 20896-1. For the validation of the printed master model, the Nikon XTH225 ST Reflection target equipment (Nikon Corporation, Tokyo, Japan) and the VGStudio MAX software (v.2023.1, Volume Graphics GmbH, Heidelberg, Germany) was used. The procedure included: positioning the sample inside the equipment, parameterizing and scanning using the CT Pro 3D (Nikon) program, followed by rebuilding the master model, importing and aligning it into VGStudio MAX, adjusting brightness and contrast to optimize image quality and making measurements. The Geometry Elements and Measurement modules were used to confirm the accuracy of the dimensions [8,16]. Finite Element Analysis (FEA) was not performed in this study; the validation procedure was limited to dimensional accuracy assessment of the printed master model using industrial CT scanning and VGStudio MAX software.

### 2.2. IOS Systems

The study protocol required standardization of scanning procedures for the two intraoral scanners evaluated. Each test object was scanned 10 times with the Trios 5 (3Shape A/S, Copenhagen, Denmark), and Medit i700 (Medit Corp., Seoul, Republic of Korea). To minimize operator-related variability and potential sources of error, all scans were performed by the same operator using consistent scanning distances and uniform movement patterns throughout the procedure [17,18].

### 2.3. Scanning Strategy

**Figure 3:**
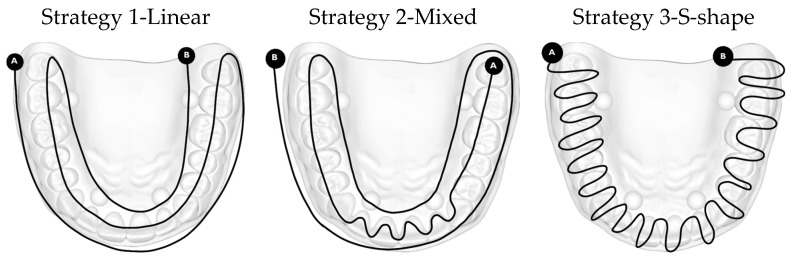
Scanning strategies analyzed in this study. Point A represents the starting point, and point B represents the endpoint of the intraoral scanning path.

The second aspect that needs to be considered and evaluated is the scanning strategies used to carry out the data acquisition. 3 scanning strategies were chosen, schematically represented in Figure 3.

The test object was positioned centrally within the acquisition area to allow an optimal scanning envelope. During scanning, the operator maintained continuous and fluid movements while preserving a consistent working distance for each intraoral scanner throughout the scanning process. The scanning distance was selected according to the operating principles and manufacturer recommendations specific to each device and was kept constant within each scanner. For the Medit i700, the working distance corresponded to the manufacturer-defined effective scanning depth, which allows reliable image acquisition within a specified focal range [19]. For the TRIOS 5 the scanner was maintained within the optimal focus range indicated by real-time optical feedback, as recommended for confocal-based intraoral scanning systems [20]. Previous studies have demonstrated that scanning distance influences trueness and precision and that optimal accuracy is achieved at scanner-specific working distances rather than at a single universal value [9]. Accordingly, the reported distance interval of approximately 5 to 30 mm reflects scanner-dependent optical characteristics and focal depth requirements and does not indicate variability within the same scanner during acquisition. Maneuvering was more demanding during changes in scanning axis, particularly when transitioning from posterior to anterior teeth.

All manufacturers provide written or embedded guides in the software application to help practitioners maintain the correct distance and avoid including surrounding tissues in the camera’s field of view, as well as to fully benefit from the scanner’s specifications. One of these guidelines designed for carrying out the practical experiments described in this article, involves a linear movement on all vestibular, occlusal and oral surfaces, starting the scan from the vestibular side of the teeth, continuing on the occlusal face and then on the oral side [21]. The tip of the intraoral scanner was positioned mostly perpendicular to the tooth surfaces; recording the axial–mesial and distal surfaces was achieved by using 45° scanning angles (the scanning angle being the angle created between the scanner camera and the recorded surface). This strategy is visually represented in Figure 3, variant 1, named the ”linear strategy”, after the movements used.

The mixed scanning strategy, achieved by using both linear and S-shaped movements (variant 2, Figure 3), was performed at a scanning angle of 45–90°. To ensure the registration of landmarks that the IOS system recognized, the process began from the occlusal surface of the cusped lateral teeth of a hemiarch, following the line of the dental arch to the anterior with the registration of the front teeth. At this level, a brushing movement was used to imprint both the incisal edge and the incisal third of the vestibular and oral faces of the front teeth. The scanning, from this point, was continued on the other hemiarch, on the occlusal surface of the molars and premolars. The exploratory pathway then followed the scanning of the vestibular and palatal surfaces of all teeth, ending with the recording of the contact points at a 90° scanning angle [21].

Another method consists of performing an S-shaped movement on the vestibular, occlusal and oral faces of each tooth, recording each tooth relatively completely at once (variant 3, Figure 3). This scanning pathway, illustrated as Strategy 3-S-shape strategy, relies on continuous, wave-like motions that follow a repetitive S-pattern along the dental arch. By maintaining a steady lateral oscillation while advancing the scanner, the images captured broad surface information without fragmenting the acquisition into isolated segments.

These described scanning strategies were correlated with the ways scanners work, taking into account the fact that Medit uses the triangulation technique, through which the reconstruction of the surfaces is realized through the geometry of the triangle between the beam and the sensor. On the other hand, the Trios 3Shape system uses ultrafast optical sectioning technology, which has the advantage of higher speed and higher precision due to the extremely fast optical sectioning mode.

The second stage of the work protocol aimed at recording the palate vault and the four calibration balls. The scanner was returned to the occlusal morphological surfaces already recorded and the surface of the palatine arch was scanned on two axes: from the right to the left hemiarch, from anterior to posterior. The last and most difficult stage was the registration of the calibration balls and the portions around them, due to the spherical and small surfaces, amplifying the degree of difficulty and the white-transparent, highly reflective color of the polymer resin from which the 3D master model was printed. The movement used was circular, around the balls, until they were completely scanned.

Regardless of the choice of strategy, the complete scanning of the calibration balls required a deviation from its trajectory, they are being recorded by a circular motion, identically performed for all three chosen scanning strategies.

All scans were performed on a phantom head, following a uniform protocol for all 10 repetitions of each scan strategy with the two scanners, thus guaranteeing consistency and complete coverage of the dental arch.

Environmental factors were rigorously controlled to maintain uniformity between scanning sessions, as follows [14,18]:

Temperature: 23 ± 1 °C, monitored with a digital thermometer calibrated according to ISO;

Humidity: 40 ± 5%, checked with hygrometer;

Lighting: 15,000 lumens, constant in rooms of about 90 m^2^; The lighting conditions were standardized due to their documented influence on intraoral scanner accuracy, as variations in ambient illumination may affect both trueness and precision depending on the scanner technology used [22].

Master model positioning: fixed on the phantom head, to prevent displacement or warping [8].

This strict approach ensured that any differences in accuracy were attributed exclusively to the intrinsic characteristics of the scanners, eliminating external influences [23], also trying to recreate the ideal working environment, similar to the one that we can achieve on a daily basis in the dental office.

### 2.4. Data Analysis

For the analysis of the scanned data, the software Medit Link–Medit Design software (v.3.1.0, Medit Corp., Seoul, Republic of Korea), selected for its ability to align and analyze digital models [24].

The overlay strategy included the use of the Alignment Mode tool, which allowed each scan to be independently aligned with the stl of the test object set as the reference model, using the Align target data separately option to optimize accuracy. Auto-alignment features have reduced the risk of manual errors.

The deviation analysis quantified the differences between the reference model–test object and the scans obtained, using Medit Design’s Deviation Display mode, which generated deviation maps and tabular ratios in micrometers (μm). Software parameters and measuring instruments have been calibrated periodically to guarantee the accuracy and reliability of the analyses.

The reference distances among the calibration spheres were acquired utilizing the measurement instruments supplied by the analytical software. The distances were computed as linear measurements between the centers of the calibration spheres and used as reference (“true”) values for further comparisons.

All scans performed with the two intraoral scanners fully complied with this standardized protocol, using the same test object. By applying a rigorous and systematic data acquisition and analysis methodology, the study aimed to obtain robust and reproducible results on the comparative performance of intraoral scanners in terms of impression accuracy (Figure 3).

### 2.5. Statistical Analysis

All statistical analyses were performed via JASP v.0.18.1 (University of Amsterdam, Amsterdam, The Netherlands), an open-source statistical program provided at no cost for academic purposes. Descriptive statistics, such as the mean, median, standard deviation (SD), variance, and root mean square error (RMSE), have been calculated to evaluate the precision and accuracy of each intraoral scanner and scanning strategy.

The Shapiro–Wilk test was applied to determine data normality, whereas Levene’s test was utilized to confirm homogeneity of variances. All datasets showed a normal distribution (*p* > 0.05), therefore justifying the use of parametric statistical tests. The impacts of the scanning approach and intraoral scanner type were simultaneously assessed using a two-way ANOVA within a factorial design, allowing the evaluation of the primary effects of each element and any interaction effects. Tukey’s post hoc test was utilized for multiple comparisons as required. A confidence level of 95% was considered for the statistical analyses. The statistical study was predominantly descriptive; hence, the results were presented through comparison measures and observable differences, without highlighting inferential significance testing or *p*-values. All figures and comparison charts were generated in Microsoft Excel (Microsoft Corp., Redmond, WA, USA) to illustrate mean deviations, RMSE, and MAD values.

## 3. Results

To prevent potential biases, all scans were conducted by the same skilled operator according to a standardized scanning methodology under controlled ambient circumstances. Each scanning approach was executed 10 times for both intraoral scanners utilizing the same reference model, and all datasets underwent a uniform alignment and deviation analysis procedure. The presented disparities indicate observable variations associated with scanning method and scanner features, rather than biases due to procedure or measurement.

The results are presented in three complementary parts: qualitative overlap analysis and scanning time assessment (Table 2 and Table 3), quantitative evaluation of scanning strategy effects for each intraoral scanner separately (Table 4 and Table 5), and direct comparison between scanners for each scanning strategy (Table 6).

The overlapping data, resulting from the alignment of the 10 scans performed for each scanning strategy, divided on the two intraoral scanners included in the study are represented by a color map based on the images of the master model (Table 2). Through a visual analysis of the color of the maps, resulting from the alignment with the Medit application, it can be seen that the Medit scanner has more uniform and stable results, presenting larger green areas. On the other hand, the color maps resulting from scanning with the Trios 5 system indicate, in addition to the differences in color homogeneity between the scanning strategies, areas with a red tint in the lateral regions, distally, on the arches, indicating from the beginning a possible lower accuracy of these areas.

Scanning times were recorded after each scan using the proprietary software of each intraoral scanner.

The main statistical parameters used and evaluated in this study are presented below [25]:

The Min. and Max. represent the largest negative deviation value–minimum and the largest positive deviation value–maximum.

The average mean and median were calculated to characterize the central trend of the data, with the mean representing the average value of all measurements, and the median indicating the value located in the middle of the ordered distribution. These measurements allow the identification of the typical deviation value for each proposed scanning strategy and for each scanner analyzed.

The mean absolute deviation (MAD) expresses the mean of the absolute deviations from the mean value and provides information on the uniformity of the results; lower values of the MAD indicate higher accuracy.

The mean square error (RMSE) is determined as the square root of the mean squares of the differences between the measured values and their mean, being an indicator sensitive to larger errors and relevant for assessing the overall accuracy of scans.

The standard deviation (SD) reflects the degree of dispersion of the values from the mean, with a low value indicating more uniform accuracy between measurements.

The variance, defined as the square of the standard deviation, describes the degree of internal variability of the data set. Higher variance indicates lower consistency and greater dependence on external factors (operator, motion, reflections, glossy surfaces, etc.).

Avg. (+) and Avg. (−) represent the average of the positive deviation values and the average of the negative deviation values, respectively.

Trueness (90 − 10)/2 was determined by calculating the difference between 90 and 10 percent, divided by two, providing a measure of fidelity by representing the distribution of values covering 80% of the central range of the data.

The 10% and 90% values correspond to the lower (10th) and upper (90th) percentiles of the deviation values, respectively.

Finally, tolerance was expressed as the percentage of deviations within an acceptable predetermined range, indicating how often the scanner’s accuracy falls within clinically permissible limits [25].

The results, both qualitative and quantitative, show that there are differences both between the way scanners record tooth surfaces through different scanning strategies, as well as between the scanning strategies themselves. The most apparent difference during the experiments was the digital recording of the calibration balls, since the spherical shape represented an impediment especially for the Trios system, which, due to the larger tip size of the scanner, required a longer time for their complete registration. Thus, the entire registration process with the Trios scanner was made more difficult, mainly due to the changes in trajectories in the mixed scanning strategies, as well as the changes in the geometry of the master model, the distal portion of the master model having disadvantages in this regard, visible in Table 2. For Medit, the registration was more truthful in terms of movements, this system being more reliable for trajectory modification, an additional help being the smaller size of the tip for registering difficult areas. At the same time, the whitish and glossy color of the resin from which the master model was made posed problems with light reflection, especially for the Medit scanner, which had difficulty recognizing areas already scanned.

**Figure 4:**
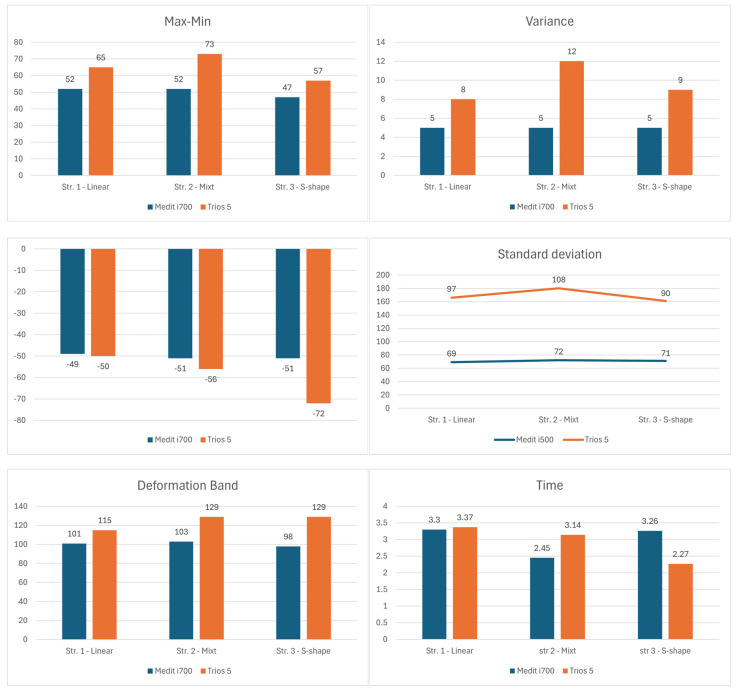
Comparative analysis of accuracy-related parameters for the Medit i700 and TRIOS 5 (expressed in micrometers (µm)), including minimum and maximum deviation values, deformation range derived from the difference between minimum and maximum values, variance, standard deviation, and scanning time.

Figure 4 provides a comparative overview of the variability and dispersion of the overlap analysis results across the evaluated scanning strategies and intraoral scanners. However, it should be noted that the differences are quite small, which shows the limitations of an in vitro study.

The deformation band, a value calculated between the minimum and maximum value of each scanning strategy, is more balanced for the Medit scanner which shows differences between 98 μm for strategy 3 and 103 μm for strategy 2 (6 μm), while the Trios scanner shows almost double differences (15 μm) from 115 μm for strategy 1 to 129 μm for strategy 3.

After analyzing the resulting data, differences were observed between the scans and the master model. The standard deviation and the variance, values that indicate the accuracy of the scans, tend to be more uniform in the case of the Medit scanner. In the case of the variance (Figure 4) the Medit scanner presents a constant value of 5 μm for all 3 scanning strategies analyzed, classifying the Medit as a stable system, regardless of the scanning strategies used. In the clinic this could be transposed through an easy use of the system, even for beginners. These results indicate that Trios 5 shows greater variability between successive measurements of the same area, especially in the mixed strategy, where the variance reaches the maximum value (12 μm). The high values of variance for the Trios 5 suggest a less constant performance between repetitions, which indicates a greater dependence on the strategy and technique of the operator. Although the scanner can achieve high levels of accuracy, these results appear to be more sensitive to changes in the scanning trajectory.

The same trend is observed through the analysis of standard deviation (SD) values, which shows a smaller dispersion of data for the Medit i700 compared to the Trios 5, regardless of the scanning strategy used. SD values for the Medit i700 range from 69 to 72 μm, indicating high consistency and superior precision of results. In contrast, the Trios 5 shows higher values between 90 and 108 μm, reflecting greater variability between measurements and thus lower accuracy.

In terms of scanning time, the differences between the two systems indicate a dependence on the strategy applied. Analyzing the average time resulting from the 10 scans performed for each scanning strategy, we observe clear differences for scanning strategies 2 and 3, between the two scanners. In the case of the mixed strategy (str. 2), the Medit i700 completes the scan faster (2.45 min) compared to the Trios 5 (3.14 min), suggesting better efficiency in capturing complex surfaces through combined movements. Strategy 3, on the other hand, offers the lowest average value obtained for the Trios scanner, which reacted faster to brush movements, indicating greater adaptability to dynamic scan strategies.

Overall, the results suggest that the Medit i700 stands out for its more consistent accuracy and reduced variability, which is confirmed by the lower values of standard deviation and variance. On the other hand, the Trios 5 demonstrates superior temporal performance in certain scanning strategies (especially the “S” type), but with a greater dispersion of data, which may indicate a higher sensitivity to the operator’s technique.

**Figure 5:**
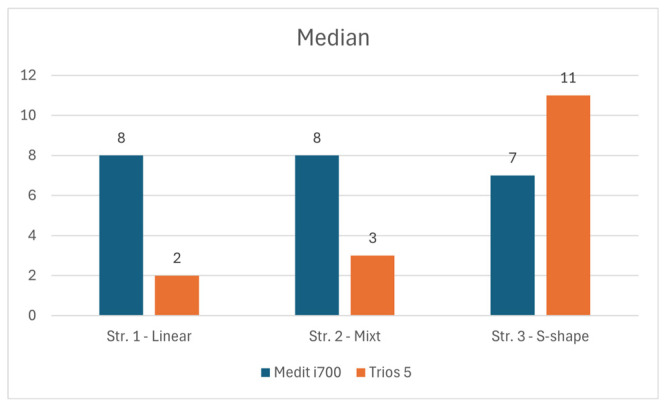

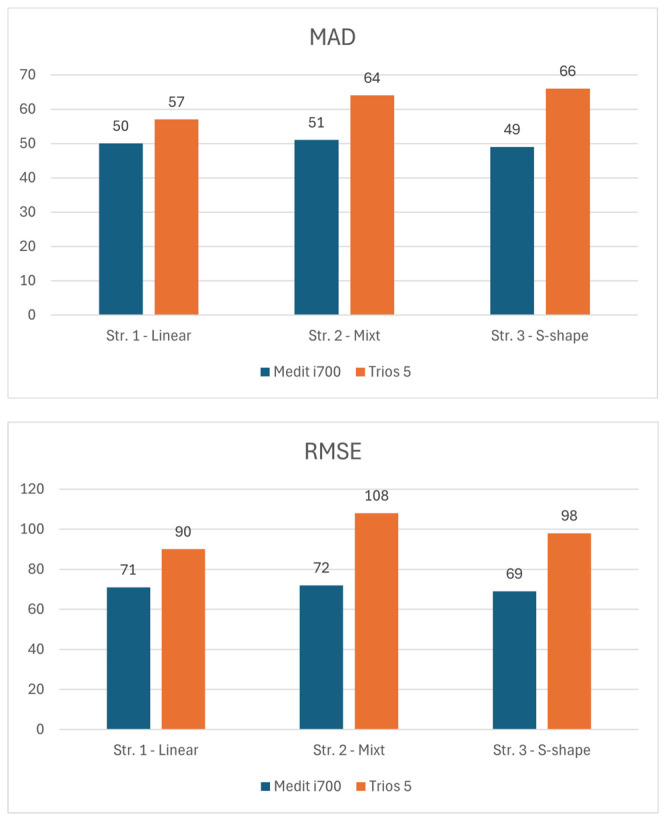
Statistical performance indices and scanning efficiency for the evaluated intraoral scanners, including median values, mean absolute deviation (MAD), and root mean square error (RMSE).

Figure 5 presents a comparative overview of the central tendency and dispersion of deviation values across the evaluated scanning strategies for both intraoral scanners.

The graph created based on median values (Figure 5), where the Medit i700 scanner showed higher values for linear and mixed strategies (8 μm), indicating a slight trend towards higher but constant mean deviations, while Trios 5 recorded lower median deviations (2–3 μm) for the same strategy, suggesting better point accuracy. However, for the S-type strategy, the Trios 5 generated a increase in the median (11 μm), which indicates a greater sensitivity to variations in the motion trajectory, while the Medit i700 maintained a relatively stable median deviation (7 μm).

From the perspective of the mean of the absolute deviations, which reflects the consistency of the values around the mean, the Medit i700 demonstrated superior stability, maintaining values between 49 and 51 μm regardless of the scanning strategy, while the Trios 5 showed a progressive increase in MAD (from 57 to 66 μm). This indicates a more pronounced variability of deviations in the case of Trios 5, especially in complex strategies, which may suggest a greater influence of the dynamics of the operator or the data processing algorithm on the consistency of the results.

As for RMSE, the global indicator of overall accuracy, the Medit i700 achieved lower values (69–72 μm), demonstrating higher overall accuracy and better ability to limit major errors. In contrast, Trios 5 recorded higher RMSE values (90–108 μm), suggesting a greater accumulation of surface errors, especially in mixed scans, a strategy that assumes greater complexity of geometry and image overlays.

## 4. Discussion

The null hypotheses were both partially rejected, as variations were observed not only between the scanning strategies but also between the two intraoral scanning systems. The first null hypothesis was partially rejected, as variations were observed between the scanning strategies and systems. The results demonstrated that the scanning strategy expresses a measurable influence on the accuracy and consistency of the digital impressions, confirming that intraoral scanning is not entirely operator-independent. Specifically, the Medit i700 exhibited superior reproducibility and lower deviation metrics (SD, MAD, RMSE), indicating a higher level of algorithmic stability and reduced sensitivity to scanning trajectory. In contrast, the Trios 5 achieved high punctual accuracy but showed greater variability depending on the regularity and continuity of the scanning path, suggesting that its data stitching and alignment algorithms may be more susceptible to interruption or angular deviation during image acquisition. These differences imply that system architecture, image capture rate, and software calibration routines play critical roles in defining scanner performance, particularly when complex or irregular scanning strategies are employed.

The second null hypothesis—that different intraoral scanners exhibit comparable accuracy—was also rejected, as clear performance differences were detected between the Medit i700 and the Trios 5. The Medit i700 displayed higher precision and lower dispersion indices (SD, MAD, RMSE), reflecting greater algorithmic stability and resistance to operator variability. Conversely, the Trios 5 demonstrated outstanding punctual accuracy but showed a higher sensitivity to deviations in the scanning trajectory, particularly in irregular or discontinuous paths. These findings suggest that although the environmental conditions were identical for the two scanning sessions, the idea arises that the differences between the two systems evaluated in the study may result due to the way the software system works, such as differences in data stitching algorithms, calibration methods, and sensor configurations, or due to human errors of the operator during the scanning process, which directly influence how each device reconstructs surface geometry, the accuracy of the final dental impression and therefore, the results of an analysis such as the one in the present study. Therefore, intraoral scanning accuracy should be considered as a dynamic interaction between system design and operator technique, rather than a fixed hardware capability.

In light of the observed values of standard deviation, variance and scan times for the Medit i700 and Trios 5, the results obtained suggest that the scanning strategy exerts an effect on the consistency and efficiency of the scanners’ performance. More specifically, the Medit i700 showed a lower dispersion of results (lower SD values and variance), which indicates more stable accuracy and less influence of operational variations between scans. In contrast, Trios 5 showed greater variability depending on strategy, although it excelled in certain strategies (e.g., the “S” strategy) in terms of scan time. This contradiction between speed and consistency reflects an inherent trade-off that the choice of scanning method can cause.

The existing literature supports the fact that the scanning strategy (scan path/scan head movement protocol) plays an essential role in determining the trueness and accuracy of digital scans. Thus, each manufacturer of IOS systems recommends an ideal scanning strategy, chosen after analyzing the technical specifications of the scanner to avoid trajectories that do not comply with the manufacturer’s recommendations, which can reduce the accuracy of scans [21]. Also, experimental studies confirm the influence of changes in the scanning trajectory on the accuracy of the full-arch impression [26]. In addition, a comprehensive analysis of the factors influencing the accuracy of intraoral scans emphasizes that the choice of scanning strategy is one of the critical parameters along with operator experience, device calibration, and environmental conditions [27,28].

Drawing a parallel with other studies comparing the Trios 5 and the Medit i700, some results indicate an advantage for the Trios 5 in terms of accuracy and trueness, while others place the Medit i700 in a competitive or even higher place, depending on the methodology and test conditions. For example, a full-arch impression study reported that the Trios 5 recorded smaller deviations for accuracy (37.8 ± 4.53 μm) and trueness (54.9 ± 11 μm), followed by the Medit i700 (accuracy: 40.6 ± 4.17 μm, trueness: 60.5 ± 10.9 μm) [29].

Therefore, interpreting our own results in the context of the literature, we can argue that the balance between scanning strategy, performance stability and scan time is a critical factor for the optimal choice of scanner and protocol. Choosing a “more aggressive” scanning strategy (e.g., S-shape paths, more complex curves) can save time, but can exacerbate stitching errors, affecting final accuracy, a phenomenon also noted in the literature [6,21,27]. The consistency and better performance of the Medit i700 indicate greater stability to variations in the operator’s technique, which can be an advantage in real clinical scenarios, where perfect repeatability is not always achievable.

The analysis of the median, MAD, and RMSE indices indicates two distinct carrier-performance relationship profiles for the two devices tested. The Medit i700 displays relatively consistent values of MAD (≈49–51 μm) and low RMSE (≈69–72 μm), signaling a more compact error distribution and a trend towards reproducible results regardless of trajectory type. Trios 5, on the other hand, exhibits higher MAD and RMSE (MAD 57→66 μm, RMSE 90→108 μm), which shows a more pronounced accumulation of errors depending on the complexity of the scanning strategy (most evident in the mixed and S-strategy). The median values support the same idea: Trios 5 offers very small deviations under simple conditions (median 2–3 μm for smooth trajectories), but the averages increase considerably in the S-shape strategy (median 11 μm), indicating a sensitivity to trajectory variations.

These observations are in partial agreement with the literature: some studies report accuracy advantages for Trios 5 in certain protocols and configurations, but also variability depending on scanning strategy and experimental conditions. For example, a recent comparative study reported better trueness values for the TRIOS 5 compared to the Medit i700 in certain experimental settings, but the differences are sensitive to the test method used [8,29]. Other previous studies have shown that the latest version of the Medit, combined with a new scanning tip, can achieve promising accuracy in full-arch digital impressions [30].

When interpreted in the context of previous literature, the deviation values obtained in the present study fall within ranges that are generally considered clinically acceptable for fully dentate digital impressions. Previous studies have reported that deviation values below approximately 50–100 µm are compatible with the accurate fabrication of tooth-supported restorations and clinically acceptable digital workflows [31,32,33,34]. Although differences between intraoral scanners and scanning strategies were observed in terms of variability and dispersion, the magnitude of the deviations recorded in this study reflects relative performance differences rather than a loss of clinical applicability under controlled conditions.

Correlating with the literature, several topics can be addressed as possible explanations for the results obtained in the present study:

### 4.1. Sensitivity to the Scanning Strategy

Meta-analyses and experimental studies constantly show that the scanning strategy (pattern followed-linear, mixed, S-shaped, etc.) [35,36,37], the presence of artificial markers and operating conditions (dry/wet) influence trueness and accuracy [6,27,38]. S-shaped patterns and the use of artificial landmarks generally reduce cumulative error. These findings suggest that the variations in RMSE and MAD observed in Trios 5 may be caused in part by the fact that this device reacts differently to changes in trajectory and the complexity of the scan path [6].

### 4.2. Alignment and Error Accumulation Algorithms

Differences in RMSE and variance between the Medit i700 and Trios 5 may reflect differences in the processing algorithms (“feature detection”, “matching”, “stitching” between frames) and in the internal calibration mode. Under conditions involving complex overlaps, such as mixed scanning strategies, the TRIOS 5 may show changes in performance consistent with previously reported error accumulation along long or irregular scanning strategies [35,39,40].

### 4.3. Concordance and Results Reported in Other Comparative Studies

Previous in vitro studies have reported variable outcomes for intraoral scanners depending on scanner type and experimental design, with some investigations describing lower mean deviation values for the TRIOS 5 during specific full-arch impression tasks and others reporting comparable or, in some cases, superior performance of Medit scanners under different experimental conditions [8,41,42]. More recent comparative studies directly evaluating the same scanner generation, the Trios 5 and Medit i700, have demonstrated largely comparable deviation patterns between the two systems. One of these studies reported clinically acceptable full-arch deviation values for both scanners (~112 µm for TRIOS 5 and ~114–117 µm for Medit i700) [16], while other similarly found marginally lower trueness and precision values for the TRIOS 5 (approximately 37.8–54.9 µm) compared with the Medit i700 (40.6–60.5 µm) [43]. In the present study, median deviation values ranged from approximately 2 to 11 µm for the TRIOS 5, depending on the scanning strategy, and between 7 and 8 µm for the Medit i700, indicating overall comparable accuracy. Differences between studies are likely attributable to variations in experimental protocols, including scanning strategy, reference model geometry, data processing workflows, and operator-related factors [8,44,45].

In addition to studies directly comparing the TRIOS 5 and Medit i700, investigations evaluating a broader range of intraoral scanners provide further context for the present findings. A recent meta-analysis confirmed that scanning strategy is a major determinant of intraoral scanner accuracy across multiple IOS systems, with some scanners demonstrating greater robustness to trajectory variations, while others achieve higher precision under optimized scanning protocols [6]. Similarly, comparative in vitro studies including multiple scanners, such as Primescan, have reported consistent trueness for Medit devices and high precision for TRIOS systems, albeit with increased sensitivity to scanning strategy and acquisition sequence. These observations are consistent with the trends identified in the present study and support the interpretation that intraoral scanner performance should be evaluated in relation to both system design and applied scanning strategy rather than as an isolated device characteristic [6,8].

### 4.4. Clinical Implications

If the clinical objective is consistency under variable conditions (different team, slightly variable technique), the present findings suggest that the Medit i700 may provide greater predictability due to low MAD/variance.

If maximum point-wise accuracy (in this context, point-wise accuracy refers to the magnitude of local deviations at individual surface points, as reflected by median deviation values) is pursued under controlled conditions (experienced operator, optimized trajectory), Trios 5 can achieve very good performance, but with an increased risk of variability when the strategy deviates from the ideal protocol.

In addition to scanner type and scanning strategy, scan time represents a clinically relevant factor that may indirectly influence digital impression accuracy. Although the present study was performed under controlled in vitro conditions, prolonged scanning times and complex acquisition protocols have been associated with a higher risk of cumulative errors, particularly in clinical environments. In such settings, operator fatigue, reduced concentration, and unintended deviations from the planned scanning path may occur. Previous research has shown that both scanning speed and scanning pattern can influence intraoral scanner accuracy and that extended or complex scanning sequences may negatively affect trueness and precision due to error accumulation [21,35]. Consequently, the differences in scanning time observed in this study should be interpreted not only from an efficiency perspective but also as a potential contributor to variability in scan accuracy during routine clinical workflows.

Clinically, these results underscore the need to align the choice of scanner and scanning strategy with the operator’s level of experience and the clinical scenario. Scanners like the Medit i700, which offer stronger internal error correction and tolerance to trajectory variation, may ensure more predictable outcomes in daily practice, while systems such as the Trios 5 may require more standardized and controlled scanning protocols to fully exploit their intrinsic accuracy. The findings further highlight the importance of operator training, scanning standardization, and software calibration in achieving consistent results across diverse clinical workflows [42,43,44,45,46].

Despite the methodological rigor of this study, several limitations must be acknowledged. The accuracy of IOS scans is strongly influenced by operator-related factors and device characteristics, making proper knowledge and skill essential for optimal performance [46]. In the present study, all scans were performed by a single experienced operator to minimize procedural variability and ensure standardized data acquisition. However, previous clinical investigations have demonstrated that operator experience affects IOS accuracy and scanning time, with increased variability observed among less experienced users [45]. Consequently, the results primarily reflect scanner performance under controlled conditions and may not fully represent outcomes obtained by operators with different levels of clinical experience.

Furthermore, the in vitro design does not fully replicate intraoral conditions such as saliva, soft-tissue movement and the use of a single operator restricts the assessment of inter-operator variability. Ambient lighting represents another critical factor affecting IOS accuracy. Previous investigations have demonstrated that variations in ambient illumination can influence both trueness and precision by altering surface reflectivity, image contrast, and the performance of optical sensors and reconstruction algorithms, with optimal lighting conditions being scanner-dependent. In addition, the extent of the scanned area may interact with lighting conditions, as larger scan spans have been associated with increased cumulative deviations, further emphasizing the importance of controlled illumination during digital impression procedures [22,47]. Moreover, the evaluation was limited to two intraoral scanners and a restricted number of scanning strategies, which may limit the generalizability of the findings to other devices or workflows. Finally, the use of a static test model and proprietary analysis software may not fully capture the complexity of clinical scanning scenarios.

Intraoral scanners (IOS) provide several key advantages, such as enhancing communication between clinicians and dental technicians, eliminating the need for traditional plaster models, and reducing overall treatment time. The accuracy and trueness of these devices are critical, as they directly influence the fit and quality of dental restorations [31,32]. However, the IOS scanning process is not free from errors, since each scan involves capturing a large number of images. Inaccuracies can arise from overlapping images during acquisition and processing, particularly in anterior teeth, which feature steep inclines and limited surface area. Additionally, software-related factors, including filter algorithms and calibration procedures, can further contribute to these deviations [33,34].

Future research should build on these findings by extending the experimental design to in vivo conditions, where dynamic soft-tissue behavior, multiple operators, and a broader range of scanning protocols can be evaluated. Including a larger and more diverse set of intraoral scanners would allow a more comprehensive assessment of current digital workflows, particularly newer generations of established systems such as the TRIOS 6, as well as scanners based on different optical concepts, including Primescan (Dentsply Sirona) and photogrammetry-enabled devices such as the Aoralscan Elite Wireless (Shining 3D). Future studies should also focus on more complex clinical scenarios, including partially and fully edentulous arches and implant-supported restorations, potentially integrating extraoral or body-scanning approaches for full-arch implant workflows. In addition, the use of advanced, high-resolution analysis software for model superimposition and deviation assessment may further refine accuracy evaluation. Together, these research directions could support the development of more robust and standardized scanning protocols and operator training strategies across a wide range of clinical applications in digital dentistry.

## 5. Conclusions

This study demonstrates that intraoral digital impression accuracy is influenced by both the scanning strategy and the intraoral scanner used. The first null hypothesis was partially rejected, as different scanning strategies resulted in measurable differences in accuracy, trueness and precision. The Medit i700 showed more consistent accuracy across all evaluated strategies, whereas the TRIOS 5 achieved lower median deviation values under certain conditions but exhibited greater variability when the scanning strategy deviated from a regular scanning path.

The second null hypothesis was also rejected, as performance differences were observed between the two scanners under identical experimental conditions. Overall, the Medit i700 demonstrated greater stability and lower dispersion of deviation values, while the TRIOS 5 showed higher point-wise accuracy under controlled scanning strategies but increased sensitivity to operator-dependent variations. These findings underline the importance of selecting both an appropriate intraoral scanner and a standardized scanning strategy to achieve reliable digital impressions.

Overall, the results indicate that both evaluated intraoral scanners can provide reliable full-arch digital impressions in fully dentate conditions when appropriate scanning strategies are applied, underscoring the importance of protocol standardization for consistent clinical outcomes.

## Figures and Tables

**Table 1:** Specifications and parameters used for the construction of the digital test object.

Parameter	Values (mm)
a–b	32
b–c	19,474
c–d	32
a–d	38
a–c	42
b–d	42

**Table 2:** Overlap analysis data obtained from ten repeated scans for each intraoral scanner (color mapping of results).

Scaner	Strategy 1	Strategy 2	Strategy 3
MEDIT	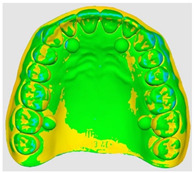	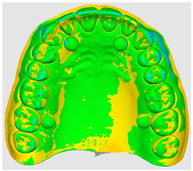	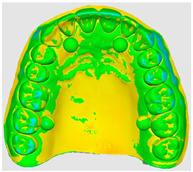
TRIOS	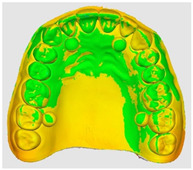	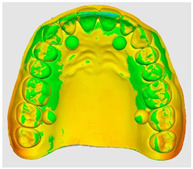	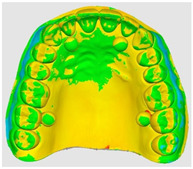

**Table 3:** The scanning times obtained for each scan, expressed in minutes and seconds (min:s).

Scan	Str. 1		Str. 2		Str. 3	
	Medit	Trios	Medit	Trios	Medit	Trios
1.	3 min 20 s	3 min 30 s	3 min 00 s	3 min 17 s	2 min 13 s	1 min 50 s
2.	2 min 54 s	3 min 26 s	2 min 23 s	2 min 33 s	3 min 15 s	2 min 19 s
3.	2 min 40 s	3 min 40 s	2 min 58 s	2 min 35 s	3 min 20 s	2 min 27 s
4.	2 min 40 s	4 min 00 s	3 min 00 s	3 min 04 s	2 min 40 s	2 min 40 s
5.	3 min 10 s	3 min 57 s	2 min 33 s	3 min 05 s	3 min 05 s	2 min 47 s
6.	3 min 26 s	4 min 00 s	2 min 45 s	3 min 06 s	3 min 05 s	2 min 30 s
7.	3 min 19 s	2 min 56 s	2 min 34 s	2 min 40 s	2 min 35 s	2 min 50 s
8.	3 min 04 s	3 min 40 s	2 min 30 s	2 min 55 s	3 min 23 s	2 min 30 s
9.	3 min 26 s	3 min 20 s	2 min 30 s	3 min 07 s	3 min 19 s	2 min 40 s
10.	4 min 00 s	3 min 04 s	2 min 00 s	2 min 45 s	3 min 05 s	2 min 37 s
Medium	3 min 30 s	3 min 37 s	2 min 45 s	3 min 14 s	3 min 26 s	2 min 27 s

**Table 4:** Results of the overlap analysis performed in the Medit Design application, showing the effect of different scanning strategies for Medit i700, expressed in micrometers (µm).

Properties	Medit i700		
	Str. 1	Str. 2	Str. 3
Min.	−1191 µm	−1983 µm	−1957 µm
Max.	1994 µm	1973 µm	1982 µm
Median	−8 µm	−8 µm	−7 µm
Average Mean	−4 µm	−5 µm	−7 µm
MAD	50 µm	51 µm	49 µm
RMSE	71 µm	72 µm	69 µm
SD	71 µm	72 µm	69 µm
Variance	5 µm	5 µm	5 µm
Avg. (+)	52 µm	52 µm	47 µm
Avg. (−)	−49 µm	−51 µm	−51 µm
(90 − 10)/2 (Trueness)	76 µm	78 µm	75 µm
10%	−80 µm	−84 µm	−86 µm
90%	71 µm	72 µm	63 µm
Tolerance	59.85%	58.80%	61.36%

**Table 5:** Results of the overlap analysis performed in the Medit Design application, showing the effect of different scanning strategies for Trios 5, expressed in micrometers (µm).

Properties	Trios 5		
	Str. 1	Str. 2	Str. 3
Min.	−1993 µm	−1990 µm	−1999 µm
Max.	1999 µm	2000 µm	1999 µm
Median	−2 µm	−3 µm	−11 µm
Average Mean	6 µm	6 µm	−15 µm
MAD	57 µm	64 µm	66 µm
RMS	90 µm	108 µm	98 µm
SD	90 µm	108 µm	97 µm
Variance	8 µm	12 µm	9 µm
Avg. (+)	65 µm	73 µm	57 µm
Avg. (−)	−50 µm	−56 µm	−72 µm
(90 − 10)/2 (Trueness)	84 µm	91 µm	100 µm
10%	−76 µm	−85 µm	−123 µm
90%	92 µm	97 µm	76 µm
Tolerance	59.38%	56.76%	51.14%

**Table 6:** Results of the overlap analysis performed in the Medit Design application, showing the differences between the two intraoral scanners for each scanning strategy, expressed in micrometers (µm).

Properties	Str. 1		Str. 2		Str. 3	
	Medit	Trios	Medit	Trios	Medit	Trios
Min.	−1191 µm	−1993 µm	−1983 µm	−1990 µm	−1957 µm	−1999 µm
Max.	1994 µm	1999 µm	1973 µm	2000 µm	1982 µm	1999 µm
Median	−8 µm	−2 µm	−8 µm	−3 µm	−7 µm	−11 µm
Average Mean	−4 µm	6 µm	−5 µm	6 µm	−7 µm	−15 µm
MAD	50 µm	57 µm	51 µm	64 µm	49 µm	66 µm
RMS	71 µm	90 µm	72 µm	108 µm	69 µm	98 µm
SD	71 µm	90 µm	72 µm	108 µm	69 µm	97 µm
Variance	5 µm	8 µm	5 µm	12 µm	5 µm	9 µm
Avg. (+)	52 µm	65 µm	52 µm	73 µm	47 µm	57 µm
Avg. (−)	−49 µm	−50 µm	−51 µm	−56 µm	−51 µm	−72 µm
(90 − 10)/2 (Trueness)	76 µm	84 µm	78 µm	91 µm	75 µm	100 µm
10%	−80 µm	−76 µm	−84 µm	−85 µm	−86 µm	−123 µm
90%	71 µm	92 µm	72 µm	97 µm	63 µm	76 µm
Tolerance	59.85%	59.38%	58.80%	56.76%	61.36%	51.14%

## Data Availability

The original contributions presented in the study are included in the article; further inquiries can be directed to the corresponding authors.
